# Working memory supports rapid talker and accent accommodation: An individual differences investigation

**DOI:** 10.3758/s13423-025-02851-x

**Published:** 2026-02-17

**Authors:** Drew J. McLaughlin, Grace E. Teuscher, Melissa M. Baese-Berk, Kristin J. Van Engen

**Affiliations:** 1 Department of Psychological & Brain Sciences, Washington University in St. Louis, St. Louis, MO USA; 2 Basque Center on Cognition, Brain and Language, Paseo Mikeletegi, 69, 20009 Donostia-San Sebastián, Gipuzkoa Spain; 3 Department of Linguistics, University of Oregon, Eugene, OR USA; 4 Department of Linguistics, University of Chicago, Chicago, IL USA

**Keywords:** Speech perception, Working memory, Talker accommodation, Accent

## Abstract

Under multitalker listening conditions, listeners appear to rapidly accommodate variability in speaker productions. However, evidence indicates that this trial-to-trial accommodation incurs a processing cost and is amplified by accent differences among talkers. The present study investigated how individual listener differences in working memory capacity and attentional control may predict the degree of processing costs (as measured by response time and accuracy) incurred by trial-to-trial switches in English talkers of first language (L1) American accent and second language (L2) Mandarin Chinese accent. Listeners completed a dual-task experiment with a primary listening task and secondary non-linguistic task as well as measures of working memory capacity (the Word Auditory Recognition and Recall Measure) and attentional control (the Trail Making Test and a Stroop Test). Results indicated that, on trials with a talker switch, listeners with smaller working memory capacities were slower to respond to the secondary task and had poorer recognition accuracy for the primary task. The measures of attentional control did not predict switching costs. One marginal interaction emerged between Stroop test scores (typically used to assess selective attention and inhibitory control) and the accent manipulation, such that listeners with better attentional control had marginally better overall recognition accuracy for L2 accent. Working memory capacity, on the other hand, did not predict differences in recognition accuracy between accent conditions. We conclude that working memory plays a critical role in supporting rapid (trial-to-trial) talker accommodation. Attentional control may support processing of L2 accent more generally but not be critical for rapid accommodation.

**Supplementary Information:**

The online version contains supplementary material available at 10.3758/s13423-025-02851-x.

Although speech processing often appears effortless, prior evidence indicates that the unique qualities of individual talkers’ productions can complicate speech processing (Luthra, 2024). For example, experiment blocks with multiple speakers, changing from trial-to-trial, tend to increase processing costs (as evidenced by poorer accuracy and increased response times) as compared with blocks with a single speaker (Choi et al., 2018; Choi & Perrachione, 2019; Heald & Nusbaum, 2014; Martin et al., 1989; Mullennix et al., 1989; however, see Saltzman et al., (2021), for some limitations of prior studies that recycled materials). Evidence from McLaughlin, Colvett, and colleagues ([Bibr CR37][Bibr CR38]) further indicates that the processing costs associated with accommodating talker variation can be examined trial-to-trial; results of their analyses revealed consistent costs for trials in which a switch in talkers had occurred, and these costs were greatest when switching from a speaker with first language (L1) accent to a speaker with second language (L2) accent. In the present study, we sought to build on prior work by investigating the potential roles of working memory and attentional control in managing trial-to-trial changes in talker and accent.

The term working memory was first coined by Miller et al. (1960) to distinguish between the passive holding of information (short-term memory) versus holding information in memory in the service of ongoing mental activity (for example, language processing). Correspondingly, working memory capacity refers to the functional capability of an individual’s system and is practically estimated in terms of the number of units of information the individual can hold in memory (while completing a cognitive process) at a given time. Attentional control, on the other hand, is defined as the individual’s ability to engage in goal-directed behavior (Posner, 1982). A variety of classic tasks, including the Stroop (1935) and Eriksen flanker (Eriksen & Eriksen, (1974), are commonly used to examine individual differences in attentional control ability.

However, separating working memory and attentional control into discrete cognitive components poses both a theoretical dilemma as well as methodological difficulties. It remains an active topic of debate as to whether working memory capacity and attentional control represent unique cognitive components. Using a factor analytic approach in a large sample of subjects across the adult lifespan, McCabe et al. (2010) found evidence of strong correlations among several tests of working memory and executive function (including attentional control). Indeed, it has been shown that attention affects performance on working memory tests, both in terms of attention allocated to maintaining items in memory and in terms of fluctuating attention across trials (Unsworth & Robison, (2015). Conversely, participants with larger working memory capacities have been found to be better at sustained attention (Unsworth et al., (2010), Eriksen flanker (Heitz & Engle, 2007), dichotic listening (Conway et al., 2001), and Stroop tasks (Kane et al., 2001). Inflating this issue, not all working memory and attentional control tasks are equal in terms of their predictive capacities (Draheim et al., 2022), and correlations among tasks meant to index the same underlying constructs, such as the Eriksen flanker and Stroop task, do not always correlate (Hedge et al., 2018).

In the present study, we use an individual differences approach to examine whether working memory and/or attentional control processes support speech processing during transitions between talkers and accents. We primarily investigate predictions stemming from the *active control model* (Magnuson & Nusbaum, 2007; Nusbaum & Magnuson, 1997). This model proposes that the costs of trial-to-trial talker changes reflect the attentional and memory load required to achieve phonetic constancy (Heald et al., 2016). More specifically, this account proposes that attentional control is necessary to support recruitment of a talker accommodation mechanism that can either:

- Map the speaker’s idiosyncratic productions to the listener’s phonological space, or
- Match the speaker’s idiosyncratic productions to representations in memory (i.e., for familiar talkers; see schematic by Magnuson, 2018; however, see also counterevidence in Magnuson et al., 2021).

Critically, the active control model also posits a key role of working memory (Nusbaum & Morin, 1992); when a change in talkers is detected, the speech signal is held in working memory while a mapping is computed (and/or a current mapping is checked for errors and refined).

An alternative account, the *auditory streaming framework*, proposes that the processing costs associated with speaker changes stem from disruption of auditory attention (Shinn-Cunningham, 2008). On this view, processing costs are incurred as a listener refocuses their attention from one auditory object (a speaker) to another (Mehraei et al., 2018). Under this framework, all transitions between talkers—regardless of how predictable an upcoming transition is—ought to impose a cost. Key evidence supporting this account stems from work by Kapadia and Perrachione (2020), in which the authors found that the number of talkers in a multitalker block did not affect the degree of processing costs; rather, a flat cost appeared to be present for any block with two or more talkers. Additionally, even when changes in talkers were completely predictable (i.e., changing back and forth between two talkers consistently) processing costs persisted. From these findings, Kapadia and Perrachione concluded that transitions between talkers incur a fixed cost because of the attention required to change between auditory objects.

Critically, the auditory streaming framework does not pose a role of working memory in accommodation of talker changes (see also Choi et al., 2022). Note also that although the auditory streaming framework proposes a key role of disruptions to attention, this is not synonymous with attentional control. Attentional control refers to an individual’s ability to engage in volitional, goal-directed behavior (e.g., by inhibiting irrelevant information), whereas the auditory streaming framework posits nonvolitional, involuntary costs stemming from talker changes.

Further work suggests that a combination of these two models may best account for speaker-switching costs. Results of McLaughlin, Colvett et al. ([Bibr CR37][Bibr CR38]) support the conclusion that multiple mechanisms may operate in parallel to support rapid accommodation. Using pupillometry, a physiological index of cognitive processing load (Beatty, 1982), the authors examined speech processing by L1 English participants during trial-to-trial changes between English speakers with L1 and L2 (Mandarin Chinese) accents. Trials in which a change in talker occurred elicited larger pupil responses during sentence processing (i.e., greater cognitive processing load) than trials in which no change occurred. Further, talker switches from an L1-accented speaker to an L2-accented speaker (“across-accent”) were more challenging than switches between two L2-accented speakers (“within-accent”). Critically, the greater processing costs observed for across-accent switches as compared with within-accent switches may indicate that two separate mechanisms support rapid talker accommodation: A mechanism that induces a processing cost when any type of change in talkers occurs (e.g., akin to an auditory streaming mechanism), and a mechanism that induces an additional cost and is specifically recruited for computing mappings to L2 accent (e.g., a talker, or accent, accommodation mechanism). While at first glance the auditory streaming framework (as described by Shinn-Cunningham, 2008) seems to describe the former mechanism, it must be noted that the design of McLaughlin, Colvett and colleagues ([Bibr CR37][Bibr CR38]) conflicts with the framework’s assumption of continuous auditory input; in McLaughlin et al., there were not only periods of silence between auditory stimuli (i.e., between trials), but also verbal repetitions by the participant. Thus, although any type of across-trial talker change did incur a cognitive cost—as would similarly be expected from disruptions to auditory streaming—this particular mechanism appears to operate on a longer timescale. It remains to be determined whether this is a distinct mechanism engaged for processing talker changes, or a more domain general process (e.g., attentional control).

In the present study, we build on McLaughlin, Colvett et al. (2024b) by examining whether individual differences in working memory capacity and attentional control are related to the processing costs associated with trial-to-trial changes between talkers of the same and different accents. Over longer (experiment-length) timescales, a substantial literature indicates that listeners can rapidly accommodate L2 accent, thereby improving the accuracy (Bradlow & Bent, 2008) and reducing the costs (Brown et al., 2020; Clarke & Garrett, 2004) of speech processing (see review by Bent & Baese-Berk, 2021). Further evidence also suggests that working memory and/or attentional control may support accommodation over longer timescales. For example, working memory is likely critical for temporary storage of the incoming speech stream, such that L2-accented productions—which potentially result in a greater number of one-to-many mappings and/or errors—can be reanalyzed (Heald & Nusbaum, 2014). Attentional control, on the other hand, may aid the listener in selection and enhancement of the most critical aspects of the speech signal, or in filtering out uninformative cues that occur frequently in L2 speech (e.g., atypical pauses within phrases; Amitay, 2009).

Evidence linking working memory and attentional control with L2 accent processing and/or adaptation remain mixed. Banks et al. (2015) examined L1 listener’s recognition accuracy for sentences produced in a novel (artificially constructed) accent across a six-block experimental session. Listener performance improved across the task at a faster rate, and to a greater degree, for individuals with better attentional control (as measured by a Stroop test). Working memory capacity (as measured with a reading span task), on the other hand, was not directly related to overall performance or adaptation. In contrast, evidence from McLaughlin et al. (2018) indicates a key role of working memory—but not necessarily attentional control—in L2 accent perception. McLaughlin et al. (2018) examined transcription accuracy by L1 listeners for semantically anomalous (but syntactically normal) sentences spoken by an L1- and an L2-accented speaker of English. The L1-accented stimuli were presented in either speech-shaped or cafeteria noise and the L2-accented stimuli were presented in either quiet or speech-shaped noise (i.e., four conditions total), and additional measures of cognitive ability were collected. Unlike Banks et al. (2015), the conditions in McLaughlin et al. (2018) were randomly intermixed, and, thus, only relationships between the cognitive measures and overall task performance (not adaptation to the accent) were examined. An analysis of individual differences indicated that listeners with larger working memory capacities performed better overall in both L2 accent conditions, but not the L1 accent (in noise) conditions; thus, working memory appeared to be critical for L2 accent perception. Attentional control (assessed with a Stroop test) was also investigated, but did not correlate with performance in any conditions.

To summarize, the cognitive costs associated with processing unfamiliar L2 accent in general (as compared with L1 accent; McLaughlin & Van Engen, 2020), and adapting to unfamiliar L2 accent over the course of an experiment, may be due to increased engagement of attentional control (Adank & Janse, 2010; Janse & Adank, 2012) and/or increased demands on working memory (McLaughlin et al., 2018). Likewise, models of the cognitive costs associated with rapid, trial-to-trial accommodation of talker changes propose recruitment of these same cognitive resources. By examining individual listener differences in working memory capacity and attentional control, the present study aimed to untangle these related topics.

## The present study

The present study used a dual-task paradigm previously used by Strand et al. (2018) and Brown et al. (2020). Dual-task paradigms leverage the concept of a limited-capacity system to investigate cognitive processing load for a given task. Sentences spoken by three L1-accented (American) talkers and three L2-accented (Mandarin Chinese) talkers were presented to L1 English listeners in a randomized order. By examining the context of the prior trial (trial *N* − 1), this design creates three conditions: No switch (the same talker on trial *N* and trial *N* − 1), within-accent switch (different talkers on trial *N* and trial *N* − 1, but both of L1 or both of L2 accent), and Across-Accent Switch (different talkers of different accents on trial *N* and trial *N* − 1). We predicted that (1) response times would be slower and speech recognition accuracy would be poorer for trials with L2 accent than L1 accent; (2) response times would be slower and speech recognition accuracy would be poorer for trials in which a switch in talkers had occurred; and (3) response times would be slower and speech recognition accuracy would be poorer for Across-Accent Switch trials than Within-Accent Switch trials (as in McLaughlin, Colvett et al., [Bibr CR37][Bibr CR38]). Note that in the context of the current study, the measure of response time comes from a dual-task paradigm designed to index cognitive processing load (as opposed to a response time index of processing speed, as in studies such as Kapadia & Perrachione, 2020). Finally, we predicted an asymmetry in across-accent switching costs matching the results of McLaughlin, Colvett et al., such that switches from L1 to L2 accent would be more cognitively demanding than switches from L2 to L1 accent.

Individual differences in working memory capacity and attentional control, and their relationships with overall performance as well as trial-to-trial switching costs, were also examined. We predicted that there would be relationships of working memory capacity and attentional control with overall performance, particularly for L2-accented trials (i.e., interactions). Additionally, we predicted that individual differences in both working memory capacity and attentional control would interact with the effect of switching, indicating a role of both in managing trial-to-trial rapid talker and accent accommodation.

The aforementioned predictions are in line with an active control model of talker and accent switching costs. Specifically, both asymmetry in across-accent switching costs (as in McLaughlin, Colvett et al., [Bibr CR37][Bibr CR38]) and roles of working memory capacity and/or attentional control would support the active control model. However, the presence of these relationships would not negate a role of a secondary mechanism (i.e., one akin to an auditory streaming mechanism that operates on a longer timescale). Thus, our primary aim in the present study was to determine the involvement of working memory and/or attentional control during talker switching, as evidence supporting the active control model.

## Methods

The current study was approved by Washington University’s Institutional Review Board.

### Participants

Young adult subjects (*M*_age_ = 19.5 years; range: 18–30 years; 36 men, 85 women) were recruited from Washington University in St. Louis’s Psychology Participants Pool. Inclusion criteria (set via demographic filters in SONA Systems) selected for L1 English speakers with normal hearing and vision (or corrected-to-normal vision). Additional criteria on the SONA listing indicated that subjects should not sign up for the study if they had extensive exposure to Mandarin Chinese (e.g., they should not speak Mandarin Chinese, have studied Mandarin Chinese, or have parents or roommates who are fluent in Mandarin Chinese). Participants who did not complete all tasks were excluded from analyses. For the dual-task dataset, we set an a priori exclusion threshold (of greater than 3,000 ms mean response times) with the aim of screening out participants who were not actively engaged; no participants exceeded this threshold or were excluded for this reason. The final sample size was *N* = 120.

### Materials

Sentences from the Semantically Normal Sentence Test (SNST; Nye & Gaitenby, 1974) were used in the present experiment. The SNST includes semantically anomalous items with four keywords each, such as “the *wrong shot led* the *farm.*” Recordings of these sentences were created in a sound-reduction booth using MOTU UltraLite-mk3 Hybrid microphone hardware and Audacity (Version 2.4.2) run on iMac (Version 10.15.7). Six young adult women Mandarin Chinese-accented speakers were recorded reading all of the semantically anomalous items. These recordings were piloted with 253 participants, for a total of approximately 10 transcriptions per item (i.e., each participant listened to only 90 items). Three Mandarin-accented speakers who were fairly well-matched on intelligibility were selected from this pilot for use in the present study. The selected speakers were estimated to be 51.8%, 53.1%, and 55.8% intelligible; the other three speakers were estimated to be 30.5%, 40.0%, and 40.3% intelligible. Given that some studies have shown that participants will “give up” when intelligibility thresholds are below 50% (Zekveld & Kramer, 2014), we opted to use the more intelligible speakers for the present study.

For the L1-accented condition, three women L1 speakers of English from the Midwestern United States were recorded. The L1 speakers were instructed to produce items at a typical speed, a slightly slower than normal speed, and a slower than normal speed. Items were then selected based on their total duration in order to match the speaking rate across the L2 and L1 speakers. The final average stimuli length for the L2 speakers was 1,790 ms, and the final average stimuli length for the L1 speakers was 1,784 ms.

### Procedures

#### Overview

Data for the present study comes from a larger longitudinal study that examined perceptual accommodation of L2 accent across multiple days (see McLaughlin, Baese-Berk et al., 2024a. Only dual-task data from Day 1 and measures of working memory capacity and attentional control collected on subsequent days are examined in the present study. Full details of the 5-day protocol can be found in McLaughlin, Baese-Berk et al. (2024a); we report only the relevant procedures here.

The speech perception task was administered on a 21.5-in. iMac (Version 10.15.7, “Catalina”) and programmed with SuperLab (Cedrus, Version 5). Audio was presented via circumaural Beyerdynamic DT 100 headphones. The measure of working memory capacity was also administered on this iMac, but was programmed with PsychoPy 2 (Peirce et al., 2019), and the measures of attentional control were both administered on paper.

#### Dual-task paradigm

**Fig. 1:**
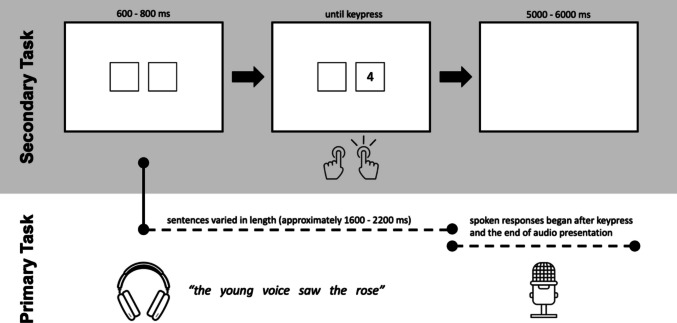
The timings of the primary (speech perception) and secondary (visual categorization) tasks are shown in parallel. All trials began with simultaneous presentation of two empty boxes onscreen and a target sentence. After a period of 600, 700, or 800 ms, the target number for the secondary task would appear. Sentences varied in length, but this timing approximately presents the target number midway through the sentence. Participants are instructed to respond via keypress on a button box as quickly as possible (i.e., they are instructed not to wait for the sentence to end). Keypresses automatically clear the screen. Once the participants are on the clear screen and have heard the entire sentence, they can begin to repeat the sentence aloud. Verbal responses are collected via microphone during the blank screen period. The screen stayed blank for a period of 5,000, 5,500, or 6,000 ms and then continued to the next trial automatically

The dual-task design, shown in Fig. 1, used in the present study was modeled after Strand et al. (2018) and Brown et al. (2020). The primary task in the dual-task paradigm examined recognition accuracy for semantically anomalous sentences and the secondary task examined nonlinguistic visual categorization. Participants were told that they would be completing both tasks simultaneously, but to prioritize the primary (speech) task over the secondary task. Each trial, subjects were presented with a single sentence over the headphones. Their goal was to repeat back the sentence at the end of the trial as accurately as possible. Simultaneously, two empty squares appeared on the screen (at the onset of the audio). After an interstimulus interval (ISI) of 600, 700, or 800 ms, a number between 1 and 8 appeared in either the left or the right box. Using a button box, participants were instructed to make either a left response or a right response depending on the following: If an odd number appeared (1, 3, 5, or 7), they were supposed to press the button on the opposite side as the box on the screen; if, however, an even number appeared (2, 4, 6, or 8) they were supposed to press the button on the same side as the box on the screen^1^. For example, the correct response for a 1 appearing in the left box on the screen was pressing the right button, and the correct response for a 2 appearing in the left box on the screen was pressing the left button. Participants were instructed to prioritize accuracy, but also respond as quickly as possible. Only trials with accurate responses to the secondary task were retained in the dataset. This resulted in the removal of 4.6% of trials in the L1 accent condition, and 5.7% of trials in the L2 accent condition.

For the primary task, participants repeated the target sentence aloud after it finished playing and their key press was made for the secondary task. Between trials, an ISI of 5,000, 5,500, or 6,000 ms occurred.

Notably, the timing of the presentation of the number for the secondary task ensured that the demands of that task occurred approximately midway through the presentation of the target sentences for the primary task. As such, the design of this dual-task paradigm ensures that the cognitive resources required to complete each task are in competition. Critically, this means that trials in which the demands of the primary task were greater should result in longer response times to the secondary task.

The combination of items for the primary and secondary tasks was randomized across participants. For the secondary task, the presentation of each number at each of the two locations was set to occur at random. For the primary task, auditory files were presented in a random order within a list used for practice trials (12 total) and a list used for regular trials (78 total). An equal number of trials for each accent condition and each speaker were included. For the regular trials, this resulted in 39 trials per accent, and 13 trials per speaker. Counterbalancing ensured that target sentences were presented in both accent conditions across subjects.

During the practice trials, a researcher remained in the room to observe the participant and confirm they were making responses in the correct order (i.e., button press and then verbal repetition). Feedback on timing of responses was included in the practice session only. If subjects took longer than 3,000 ms to respond with a button press after presentation of the number target, “Too slow!” appeared onscreen. Data from practice trials was excluded from analyses.

#### Working memory capacity

The Word Auditory Recognition and Recall Measure (WARRM) was used to assess working memory capacity (Smith et al., 2016). This task consisted of five blocks, each containing up to five trials. The difficulty (i.e., number of items) increased each block, beginning with sets of two words in the first block up to a set of six words in the last block. Each trial, participants listened to a word and were instructed to repeat it aloud. Immediately after, they judged whether the first letter of the word belonged to either the first or second half of the alphabet (spoken aloud as “first” or “second”). After all the words in a given trial had been heard, the participant had to recall each word in the order in which they heard them. If the participants could accurately recall every word in the trial, regardless of order or accuracy of the first/second judgment, they passed the trial. When they had passed three of the five trials, they passed the block and could move on to the next (more difficult) block. If they did not pass at least three of the five trials, however, the task ended. A participant’s score (i.e., estimated working memory capacity) was the highest level they passed, plus any partial credit from a higher level. For example, if a participant passed level five, and successfully completed two of the three trials necessary to pass level six, their score on the test would be 5.66 (5 + 2/3; see Smith et al., 2016). Given the robust predictive capacity of WARRM (Smith et al., 2016) as well as time constraints of the study, we did not opt for multiple measures of working memory.

#### Attentional control

Two separate tasks were used to assess individual differences in attentional control: the Trail Making Test (Bowie & Harvey, 2006) and a Stroop test (Jensen & Rohwer, 1966). We opted to include two attentional control tasks given the variable predictive capacities of attention measures in prior work (Draheim et al., 2022). For the Trail Making Test, participants completed two blocks. In Part A, an 8.5 × 11 printed page of numbers from 1 to 25, distributed pseudorandomly, was presented. Participants were instructed to draw connecting lines between the numbers in order. The time it took to complete the task was recorded with a stopwatch by the researcher. In Part B of the Trail Making Test, participants had to draw connecting lines between pseudorandomly distributed numbers and letters in the correct increasing order, alternating between the numbers (1 to 13) and the letters (*A* to *L*). The time it took to complete Part B served as the estimate of attentional control.

A paper version of the Stroop test was administered. Participants completed three sections of the test: Words, Colors, and Color-Words. For each section of the Stroop test, the participant had 45 s to read aloud as many items (i.e., words in the Words section, colors in the Colors and Color-Words sections) as possible. At the end of the allotted time, the number of words the participant read was recorded. In the Words portion of the task, the words were printed in black and white ink. For the Colors portion of the task, the letters “XXX” were printed in color ink, presenting no conflict between ink color and text. In the Color-Words portion of the task, the ink color and the written color were mismatched, e.g., “red” was printed in green ink, and the participant was instructed to ignore the printed word and read aloud the color of the ink. A single Stroop interference score per subject was calculated from performance on these three sections of the test using the formula:

$$\mathrm{CW}-\left[\left(\mathrm{W}\times \mathrm{C}\right)/\left(\mathrm{W}+\mathrm{C}\right)\right]$$

where CW is the Color-Words score, W is the Words score, and C is the Colors score (Scarpina & Tagini, 2017). This formula captures the number of Color-Words items completed while controlling for an individual’s speed in the Words-only and Colors-only sections. Higher scores from this metric indicate better attentional control.

### Analysis

#### Data preparation

Throughout the Analysis and Results sections, response time data refer to those taken from the secondary task, whereas recognition accuracy data refer to those taken from the primary task; accuracy of the secondary task is not analyzed.

Outliers in the response-time dataset were defined using Mean Absolute Deviations (MAD). Data was grouped by subject and condition to calculate MAD values, and then any trials with a response time outside of 3 MAD of the median were excluded; a total of 3.48% of trials were removed during this process. Trials in which an incorrect response to the secondary (response time) task was made were also excluded (1.74% of trials). Finally, values were log-transformed.

The same process was completed for the recognition accuracy dataset, but no trials were flagged for removal.

#### Assessing independence of memory and attentional control measures

We began by checking for correlations among the three measures of memory and attentional control (Table 1). Our a priori plan was to create an attentional control composite from the Trail Making Test scores and Stroop scores. However, participant scores on the two tasks were not significantly correlated (*r* = −.04, *t* = −0.44, *df* = 118, *p* = .66). When comparing the WARRM scores against the two attentional control measures, a significant correlation emerged with the Trail Making Test scores (*r* = −.34, *t* = −3.87, *df* = 118, *p* < .001) but not the Stroop scores (*r* = .18, *t* = 2.02, *df* = 118, *p* < .05^2^; note that after Bonferroni-correction this trend is nonsignificant). The significant negative correlation between WARRM scores and Trail Making Test scores indicates that listeners with greater working memory capacity also had greater attentional control.

**Table 1:** Correlation matrix of working memory and attentional control measures

	Trail Making	Stroop
**WARRM**	−0.34*	0.18
**Trail Making**		−0.04

*Note.* After Bonferroni multiple-comparisons correction, the alpha level for determining significance is *p* = .017. WARRM: Word Auditory Recognition and Recall Measure

Next, we examined the variance inflation factors (VIFs) with the “vif” function from the *car* package (Fox et al., 2007) in R (Version 4.0.4; R Core Team, 2021). VIFs can prove informative for determining whether correlations among variables in a model may be producing issues of multicollinearity. As shown in Table 2, the VIFs for the individual difference measures did not exceed 1.17; these VIF values are well-below typical threshold values that would indicate multicollinearity issues (e.g., 5 or 10; Craney & Surles, 2002).

**Table 2:** Variance inflation factors (VIFs)

Fixed Effect	VIF
Accent	1.00
Switch	1.00
WARRM	1.17
Trail Making	1.13
Stroop	1.04

*Note.* WARRM: Word Auditory Recognition and Recall Measure

With these correlation values and VIFs in mind, for the present study we opted to keep the three measures of individual differences separate. Although there is some evidence of shared variance between the WARRM and Trail Making Test, this relationship does not pose an issue of multicollinearity. Additionally, by keeping these predictors independent in the models we were able to determine whether they capture unique sources of variance that may differentially predict talker and accent switching costs.

#### Creation of switching conditions

By examining the context of talker and accent on the previous trial (trial *N* − 1), the design creates three conditions: no switch (the same talker on trial *N* and trial *N* − 1), within-accent switch (different talkers on trial *N* and trial *N* − 1, but both of L1 or both of L2 accent), and across-accent switch (different talkers of different accents on trial *N* and trial *N* − 1). Notably, because the six talkers were presented in true random order, the proportion of trials corresponding to each of these conditions is not equal; no switch trials account for 15% of the data, within-accent switch for 35%, and across-accent switch for 50%. The differences in frequency of each condition is a limitation that must be considered when interpreting the data.

#### Modeling procedures

For the response-time data, linear mixed-effects regression was implemented with the “lmer()” function from the *lme4* package (Bates et al., 2015) in R (Version 4.0.4; R Core Team, 2021). Random effects included random intercepts by subject and by item, and random slopes of accent by subject; random slopes of Switch were attempted but prevented model convergence and eventually removed. For the recognition accuracy data, the “glmer()” function from the *lme4* package was used, specifying a logit link function. Recognition accuracy was treated as a grouped binomial, meaning that models predicted performance using two columns of data (number of correct words, number of incorrect/missed words); this maintains the structure of the data such that each row of the dataset corresponds to a sentence with four key words while also accurately representing the basis of the data as binomial. Random effects included random intercepts by subject and by item; random slopes of accent and switch were attempted but prevented model convergence and eventually removed. Likelihood ratio tests were conducted to determine the significance of effects of interest, and *p* values for model parameters were estimated using the *lmerTest* package (Kuznetsova et al., 2017).

Fixed effects in the models included: Accent (dummy-coded as: L1 Accent [reference level], L2 Accent), Switch (dummy-coded as: No Switch [reference level], Within-Accent Switch, Across-Accent Switch), WARRM, Trail Making Test, Stroop Test, and the interactions of Accent and Switch independently and jointly with WARRM, Trail Making Test, and Stroop Test. The effects of WARRM, the Trail Making Test, and the Stroop Test were all treated as numeric predictors; all were centered and scaled with the *scale* function in R (to facilitate model convergence).

## Results

### Response time

**Fig. 2:**
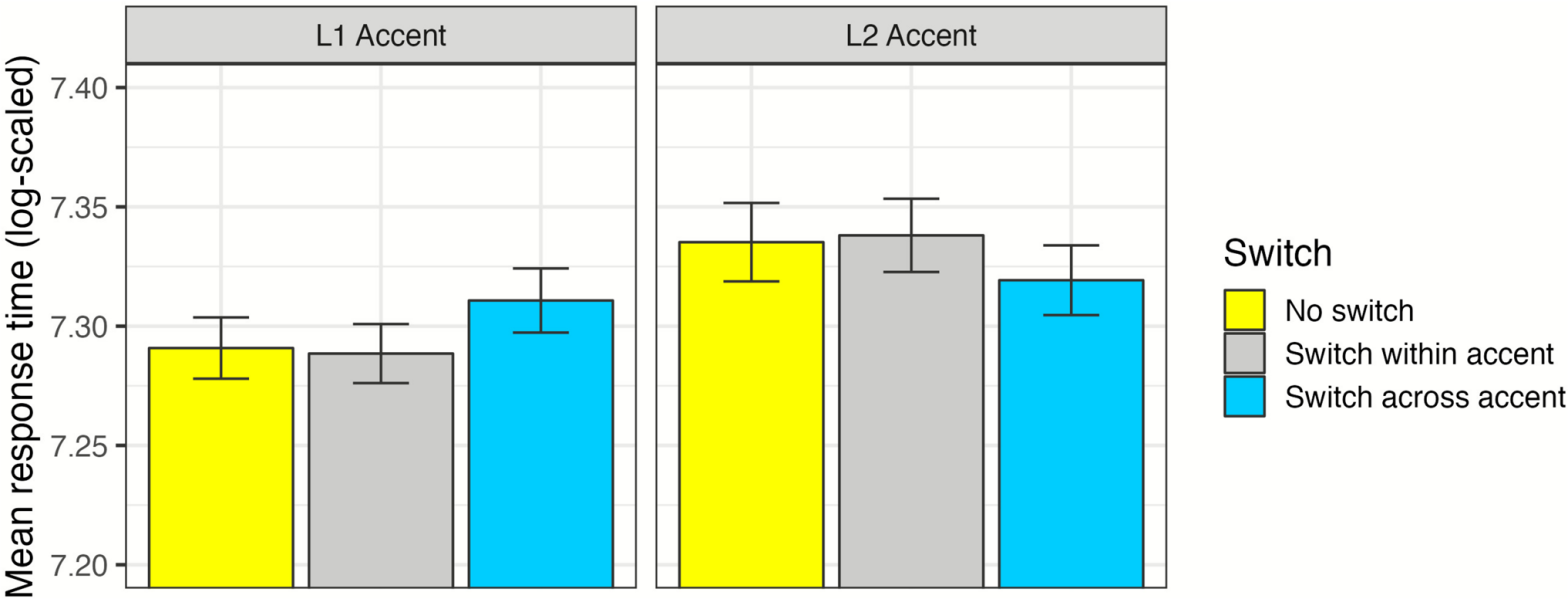
Response-time data for the interaction of Accent and Switch is presented with bars and standard errors. *Y*-axis has been truncated from 7.2 to 7.4 log-scaled units for presentation purposes. (Color figure online)

Log-likelihood model comparisons are reported in Table 3, and a summary of the maximal model is reported in Appendix A. The effect of Accent indicated that listeners were slower to respond to the secondary task for L2 Accent trials than the L1 Accent trials, as predicted (*ß* = 0.03; *χ*^2^ = 35.93, *df* = 1, *p* < .001). The effect of Switch did not improve model fit (*χ*^2^ = 0.29, *df* = 2, *p* = .86), although it did interact significantly with the effect of Accent (*χ*^2^ = 48.53, *df* = 2, *p* < .001). As shown in Fig. 2, Across-Accent Switch trials were more challenging than No Switch trials in the L1 Accent condition, but not the L2 Accent condition (interaction estimate of *ß* = −0.04, *p* < .001). Response times for Within-Accent Switch trials were similar in both accent conditions (*ß* < 0.01, *p* = .68).

**Table 3:** Log-likelihood model comparisons for response time analysis

Effect	χ^2^	*df*	*p*
Accent	35.93	1	< .001
Switch	0.29	2	.86
WARRM	6.09	1	.01
Trail Making	5.07	1	.03
Stroop	1.05	1	.31
Accent : Switch	48.53	2	<.001
Accent : WARRM	1.17	1	.28
Accent : Trail Making	0.85	1	.36
Accent : Stroop	<0.01	1	.98
Switch : WARRM	3.92	2	.14
Switch : Trail Making	0.55	2	.76
Switch : Stroop	0.09	2	.96
Accent : Switch : WARRM	3.46	2	.18
Accent : Switch : Trail Making	0.57	2	.75
Accent : Switch : Stroop	2.18	2	.34

*Note.* Colons indicate interactions. Levels of Accent included L1 Accent (reference) and L2 Accent. Levels of Switch included No Switch (reference), Within-Accent Switch, and Across-Accent Switch. WARRM: Word Auditory Recognition and Recall Measure

**Fig. 3:**
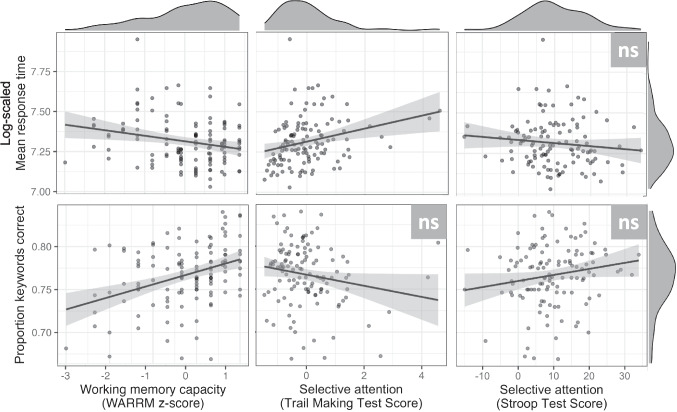
Relationships of the three *z*-scored cognitive measures of working memory capacity (i.e., the Word Auditory Recognition and Recall Measure; WARRM) and attentional control (i.e., the Trail Making and Stroop tests) with the measures of performance are presented with scatter plots and linear model fit lines with standard error. Distributions of individual subjects’ means are shown at top and right. Log-scaled mean response time values (top plots) come from the secondary (nonlinguistic) task, and mean proportion keywords correct values (bottom plots) come from the primary speech perception task. Significance was determined based on log-likelihood model comparisons; nonsignificant relationships are labeled *ns*

**Fig. 4:**
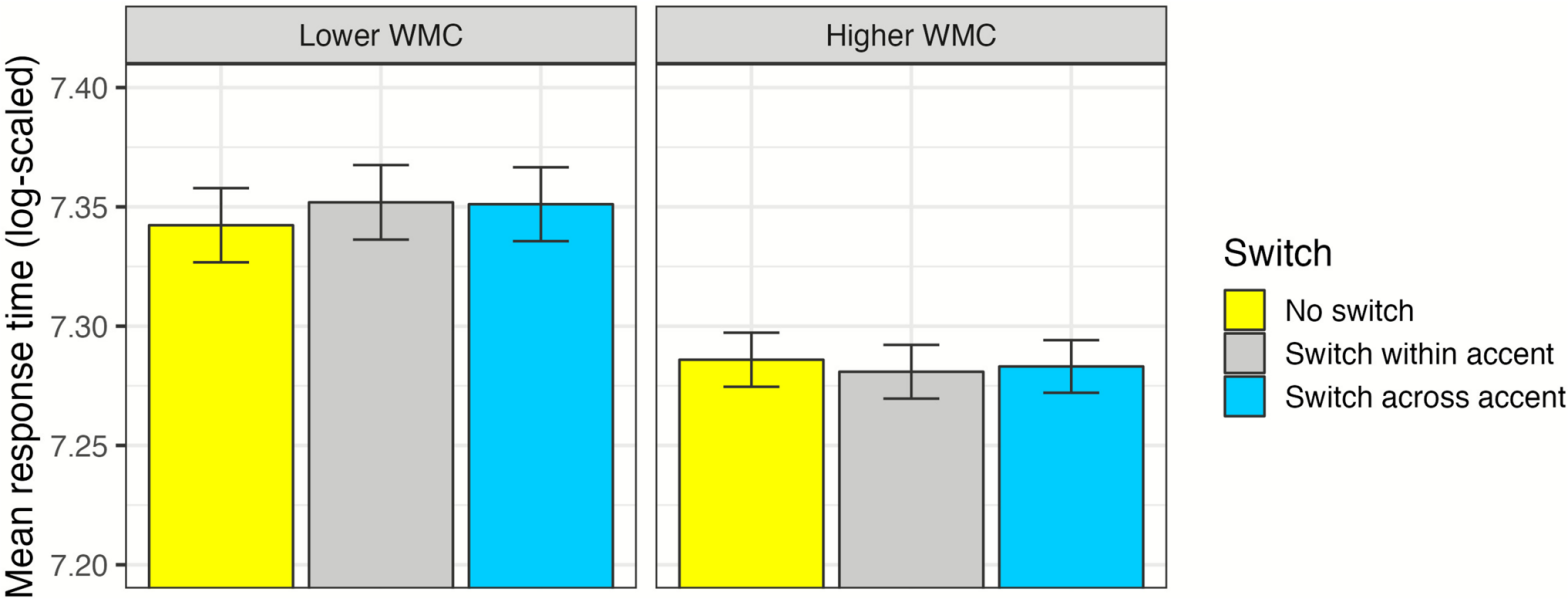
Response-time data for the interaction of working memory capacity (WMC) and Switch is presented with bars and standard errors. For visualization purposes only, subjects have been split into two groups (lower WMC versus higher WMC, split at the mean value 4.75); all analyses treated WMC as a continuous predictor. *Y*-axis has been truncated from 7.2 to 7.4 log-scaled units for presentation purposes. (Color figure online)

Both the measure of working memory capacity (WARRM) and the measure of attentional control obtained from the Trail Making Test were related to overall performance on the task (*p* = .01 and *p* = .03, respectively). Figure 3 shows the relationship between each of the three cognitive measures and overall task performance. Model estimates indicated the listeners with larger working memory capacity and better attentional control (as measured by the Trail Making Test) had faster response times. Stroop test scores, on the other hand, did not improve model fit (*χ*^2^ = 1.05, *df* = 1, *p* = .31). Of these measures, only working memory capacity showed a relationship with the effect of Switch (other *p *values > .05). Although the overall interaction was not significant (likelihood ratio test: *χ*^2^ = 3.92, *df* = 2, *p* = .14), model estimates indicated an effect of working memory capacity on the difference of Within-Accent versus No Switch conditions (*ß* = −0.01, *p* < .05); thus, participants with larger working memory capacities had somewhat smaller switching costs in some cases (see Appendix Table A2). The interaction between working memory and Switch is visualized in Fig. 4; although WARRM scores were treated as a continuous variable in the model, for visualization purposes we divide participants at the mean to create a higher working memory capacity (WMC; upper half of participants) versus lower WMC (bottom half of participants) group. Listeners with smaller working memory capacities had slower response times on Within-Accent Switch trials, as compared with No Switch trials, whereas listeners with larger capacities showed little difference.

None of the cognitive measures significantly interacted with the effect of Accent (all *p* values > .05). Lastly, we examined three-way interactions between the effects of Accent, Switch, and each cognitive measure. None of the three-way interactions improved model fit (all *p* values > .05).

### Recognition accuracy

**Fig. 5:**
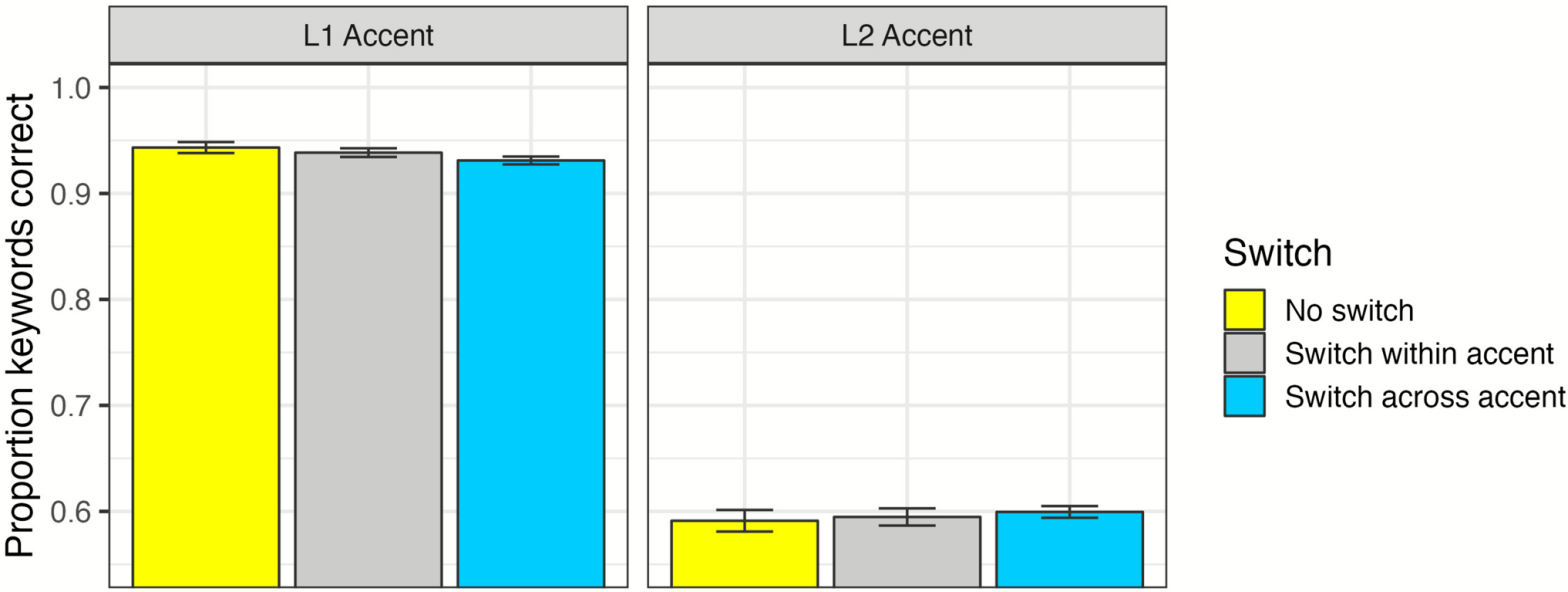
Recognition accuracy for the interaction of Switch and Accent is presented with bars and standard errors. *Y*-axis has been truncated from 0.55 to 1.00 for presentation purposes. (Color figure online)

For the analyses of recognition accuracy, log-likelihood model comparisons are reported in Table 4, and a summary of the maximal model is reported in Appendix B. The effect of Accent indicated that listeners were less accurate at repeating back keywords on trials with L2 Accent, as compared with L1 Accent (*ß* = −2.45; *χ*^2^ = 6537.40, *df* = 1, *p* < .001). The overall effect of Switch was not significant (*χ*^2^ = 1.00, *df* = 2, *p* = .61), but the interaction of Switch and Accent was marginally significant (*χ*^2^ = 5.93, *df* = 2, *p* = .05). As shown in Fig. 5, performance for Within-Accent (*ß* = −0.86, *p* = .002) and Across-Accent (*ß* = −0.57, *p* = .03) Switch trials was poorer than No Switch trials in the L1 Accent condition, in particular (Appendix Table B2). When comparing the L2 Accent condition to the L1 Accent condition, model interactions indicated slightly (but nonsignificantly) poorer performance in the for the Within-Accent Switch trials than the No Switch trials (*ß* = −0.10, *p* = .39), and slightly (but nonsignificantly) better performance in the Across-Accent Switch trials than the No Switch trials (*ß* = 0.09, *p* = .38).

**Table 4:** Log-likelihood model comparisons for recognition accuracy analysis

Effect	χ^2^	*df*	*p*
Accent	6537.40	1	<.001
Switch	1.00	2	.61
WARRM	12.03	1	<.001
Trail Making	0.86	1	.35
Stroop	1.71	1	.19
Accent : Switch	5.93	1	.05
Accent : WARRM	2.81	1	.09
Accent : Trail Making	2.12	1	.15
Accent : Stroop	3.69	1	.05
Switch : WARRM	13.20	2	.001
Switch : Trail Making	5.17	2	.08
Switch : Stroop	0.42	2	.81
Accent : Switch : WARRM	5.89	2	.05
Accent : Switch : Trail Making	0.64	2	.73
Accent : Switch : Stroop	0.33	2	.85

*Note.* Colons indicate interactions. Levels of Accent included L1 Accent (reference) and L2 Accent. Levels of Switch included No Switch (reference), Within-Accent Switch, and Across-Accent Switch. WARRM: Word Auditory Recognition and Recall Measure

**Fig. 6:**
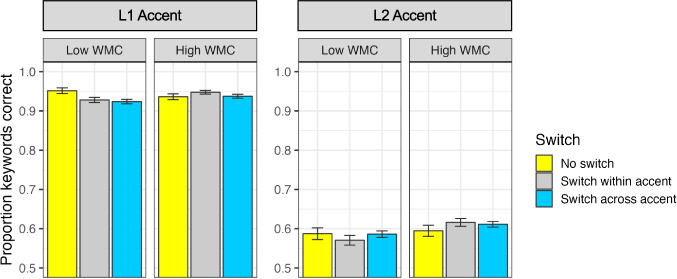
Recognition accuracy for the interaction of working memory capacity (WMC) and Switch is presented with bars and standard errors. For visualization purposes only, subjects have been split into two groups (lower WMC versus higher WMC); all analyses treated WMC as a continuous predictor. *Y*-axis has been truncated from 0.50 to 1.00 for presentation purposes. (Color figure online)

Of the three individual differences measures, only working memory capacity (*ß* = 0.09; *χ*^2^ = 12.03, *df* = 1, *p* < .001) was related to overall performance on the task; listeners with larger capacities had better overall performance (see Fig. 3). Scores from the attentional control measures did not improve model fit (both* p* values > .05). There was a significant two-way interaction of working memory capacity with the effect of Switch (*χ*^2^ = 13.20, *df* = 2, *p* < .001), and a marginal three-way interaction of working memory capacity with the effects of Switch and Accent (*χ*^2^ = 5.89, *df* = 2, *p* = .05). Figure 6 visualizes these interactions; listeners with smaller capacities had poorer performance on Switch trials as compared with No Switch trials (particularly for the L1 Accent condition), whereas listeners with larger capacities performed similarly or even slightly better on Switch trials as compared with No Switch trials.

**Fig. 7:**
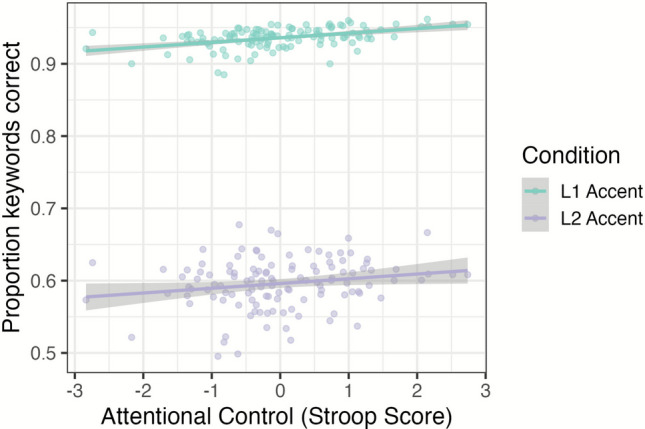
Recognition accuracy for the marginal interaction of attentional control (as measured by a Stroop task) and Accent is presented with individual participant mean points, predicted fit lines, and standard error ribbons. (Color figure online)

Of the individual difference measures, the Stroop measure of attentional control marginally interacted (*χ*^2^ = 3.69, *df* = 1, *p* = .05) with the effect of Accent (other *p* values > .05). This interaction indicated that listeners with larger Stroop scores (better attentional control) had better recognition accuracy for L2 accent overall (Fig. 7). Lastly, we examined the three-way interactions of Accent, Switch, and the two attentional control measures of individual differences, but neither improved model fit (both *p* values > .05).

## Discussion

Prior work indicates that changing trial-to-trial between talkers incurs a processing cost (Kapadia & Perrachione, 2020) and that this cost is exacerbated by accent differences among talkers (McLaughlin, Colvett et al., [Bibr CR37][Bibr CR38]). Using a multitalker dual-task paradigm, the present study examined processing costs and listening performance as a function of talker accent and trial-to-trial talker switching, as well as how individual listener differences in working memory and attentional control may account for these relationships.

Unlike McLaughlin, Colvett et al. ([Bibr CR37][Bibr CR38]), we did not find an overall cost of trial-to-trial talker switches. In both the analyses of response times (indexing processing load) and recognition accuracy, the overall trends of No Switch, Within-Accent Switch, and Across-Accent Switch trials remained similar. The interaction of Switch and Accent, however, did reveal a significant relationship: Switching costs were greater in the L1 Accent condition than the L2 Accent condition. More specifically, trials in which a switch from an L2- to an L1-accented talker occurred were most challenging. This trend is the opposite of the findings of McLaughlin, Colvett et al. ([Bibr CR37][Bibr CR38]), in which switches from L1- to L2-accented talkers resulted in the largest pupil response (indexing greater cognitive demands).

One possible explanation for this diverging outcome may be the increased difficulty of the L2 Accent condition in the present study. While McLaughlin, Colvett et al. ([Bibr CR37][Bibr CR38]) specifically examined highly intelligible speech (i.e., only trials in which the subject accurately perceived the target sentence), intelligibility in the present study was dramatically lower. For the L2 accent, recognition accuracy of keywords across all sentences was only approximately 60%; for the L1 accent, recognition accuracy was slightly below ceiling at approximately 95%. This reduced level of intelligibility was an intentional aspect of the design related to the aims of a larger accent training study from which the current study’s data comes (McLaughlin, Baese-Berk et al., 2024a). Thus, it is possible that trial-to-trial switching costs were less observable within the highly-challenging L2 Accent condition due to the reduced availability of cognitive resources (which may impede the listener’s detection of talker/accent changes). Indeed, Within-Accent Switch costs within the L2 Accent condition (i.e., switching from one L2 talker to another L2 talker) were also reduced as compared with within the L1 Accent condition (i.e., switching from one L1 talker to another L1 talker), supporting this conclusion. Examining the potential effect of task difficulty (while holding other factors, like talker accent, constant) in future research will be critical for determining whether overall task difficulty can impact the magnitude of trial-to-trial switching costs.

Based on an *active control* account, we predicted that working memory and attentional control would be critical for rapid talker and accent accommodation. This account proposes a talker accommodation mechanism that relies on working memory to hold the speech signal while a mapping is computed via attentional control processes (and/or a current mapping is checked for errors and refined). Notably, however, involvement of one or both of these cognitive resources would not rule out the possibility that multiple mechanisms are operating in parallel (see Choi et al., 2022, for evidence in favor of two mechanisms, as well as discussion of how these mechanisms’ timescales may differ). Thus, evidence supporting the conclusion that working memory or attentional control are recruited for managing talker switches would positively implicate the active control model, but not rule out involvement of either (1) a mechanism akin to that proposed in an auditory streaming framework (Shinn-Cunningham, 2008), though operating on longer timescales, or (2) a domain general attentional control process engaged when any type of change in talkers occurs.

Only individual differences in working memory capacity were related to costs of talker-switching, such that listeners with smaller capacities were slower and less accurate on Switch trials than No Switch trials. This finding aligns with an active control account of rapid (trial-to-trial) talker accommodation, in which the speech signal is held in working memory while a mapping is computed from the speaker’s idiosyncratic productions to the listener’s phonological space (Magnuson & Nusbaum, 2007; Nusbaum & Magnuson, 1997). It appears that listeners with smaller capacities had larger processing costs because they struggled to hold the target sentence in working memory during this process.

Surprisingly, neither measure of attentional control was significantly related to the costs of talker-switching. We encourage caution, however, in interpreting this lack of evidence as indicative that attentional control is not involved in rapid talker accommodation. Not all working memory and attentional control tasks are equal in terms of their predictive capacities (Draheim et al., 2022), and correlations among tasks meant to index to the same underlying constructs, such as the Eriksen flanker and Stroop task, do not always correlate (Hedge et al., 2018). It is possible that a relationship could be found using a different measure of attentional control with greater predictive validity. Indeed, one surprising finding in the present study was that individual scores on the two attention measures (the Trail Making Test and a Stroop test) were uncorrelated. This outcome suggests that each captured a unique source of variance as related to individuals’ attentional ability. For example, scores from the Trail Making Test were related to overall performance (response times) in the dual-task paradigm, but did not interact with the effect of accent. Thus, Trail Making Test scores may have primarily captured participants’ ability to manage two tasks simultaneously, rather than accent-related processing costs.

Scores from the Stroop test, on the other hand, marginally interacted with the effect of accent; listeners with better attentional control (larger Stroop scores, in this case) also had marginally better recognition accuracy for L2-accented sentences overall. The relationship between Stroop scores and performance for L2 accent in the present study is similar to the results of Banks et al. (2015), in which listener performance improved across the task at a faster rate, and to a greater degree, for individuals with better Stroop test scores (cf. McLaughlin et al., 2018). It is possible that attentional control supports L2 accent processing by inhibiting lexical competitors that wouldn’t typically be activated by L1 accent productions. For example, research examining speech reductions suggests that competitors are initially activated based on the phonological surface form of a production (Brouwer et al., 2012). Similarly, when perceiving an L2-accented production, one can expect that a different and/or larger set of lexical competitors will be activated as compared with when perceiving an L1-accented production. Results of the present study suggest that listeners with better attentional control may be better at resolving lexical competition specific to L2 accent perception.

## Conclusion

Under multitalker listening conditions, listeners appear to rapidly accommodate variability in speaker productions, including those stemming from accent differences. These trial-to-trial accommodations, however, incur a processing cost. The present study investigated how individual listener differences in working memory capacity and attentional control may be related to the degree of processing costs incurred by trial-to-trial switches of talkers with L1 and L2 accent. Our results indicate a key role of working memory in managing talker changes: On trials with a talker switch, L1 English listeners with smaller working memory capacities showed greater performance deficits (i.e., slower dual-task response times and poorer speech recognition accuracy). Our measures of attentional control were not related to switching costs, but Stroop test scores did interact with the accent manipulation such that listeners with better attentional control had marginally better overall recognition accuracy for L2 accent. Working memory capacity, on the other hand, was not related to differences in recognition accuracy between the L1 and L2 accent conditions. From these results, we conclude that working memory plays a critical role in supporting rapid (trial-to-trial) talker accommodation. Attentional control appears to support processing of L2 accent more generally. The role of working memory in rapid talker accommodation is in line with an active control model of multitalker speech processing.

## Supplementary Information

Below is the link to the electronic supplementary material.

Supplementary file1 (DOCX 24 KB)

## Data Availability

The datasets generated during and/or analyzed during the current study are available in the Open Science Framework repository (https://osf.io/c5tkx/files).

## Footnotes

1 Exploratory analyses confirmed that the number type (odd versus even) and its interaction with our manipulation were nonsignificant. We do not examine this effect further.

2 Exact *p*-values are reported to the second decimal place throughout the Analysis and Results unless below *p* < .001. Cases of “*p* < .05” denote raw values that would round up to .05, but are in fact below the significance threshold (such as .049).

## Code availability

The code used to analyze data from the current study are available in the Open Science Framework repository (https://osf.io/c5tkx/files).
