# Supplementary material for: Working memory supports rapid talker and accent accommodation: An individual differences investigation
Source: Psychon Bull Rev. 2026 Feb 17;33(3):71. doi: 10.3758/s13423-025-02851-x (PMC12913305; doi:10.3758/s13423-025-02851-x)
Supplement: Supplementary file 1 — Supplementary file1 (DOCX 24 KB) [file 13423_2025_2851_MOESM1_ESM.docx]

**Appendix A**

*Models from Analysis of Response Time (Secondary Task) Data*

**Table A1**

*Model with All Lower-Order Fixed Effects*

| **Predictor** | **Coefficient (𝛽)** | **S.E.** | ***t*** | ***p*** |
| --- | --- | --- | --- | --- |
| Intercept | 7.46 | 6.21E-02 | 120.25 | < .001 |
| Accent (L2 Accent) | 2.83E-02 | 4.39E-03 | 6.45 | < .001 |
| Switch (Within-Accent Switch) | 1.24E-03 | 4.55E-03 | 0.27 | .78 |
| Switch (Across-Accent Switch) | 2.24E-03 | 4.33E-03 | 0.52 | .60 |
| WARRM | -3.22E-02 | 1.30E-02 | -2.47 | .01 |
| Trail Making | 2.63E-02 | 1.18E-02 | 2.23 | .03 |
| Stroop | -1.29E-03 | 1.28E-03 | -1.01 | 0.32 |

**Table A2**

*Model with All Lower-Order Fixed Effects and Two-Way Interactions*

| **Predictor** | **Coefficient (𝛽)** | **S.E.** | ***t*** | ***p*** |
| --- | --- | --- | --- | --- |
| Intercept | 7.39 | 7.01E-02 | 105.38 | < .001 |
| Accent (L2 Accent) | 1.98E-02 | 2.57E-02 | 0.77 | .44 |
| Switch (Within-Accent Switch) | 5.02E-02 | 2.60E-02 | 1.93 | .05 |
| Switch (Across-Accent Switch) | 5.82E-02 | 2.47E-02 | 2.36 | .02 |
| WARRM | -1.82E-02 | 1.47E-02 | -1.23 | .22 |
| Trail Making | 3.20E-02 | 1.33E-02 | 2.41 | .02 |
| Stroop | -1.35E-03 | 1.45E-03 | -0.93 | .35 |
| Accent (L2 Accent) : Switch (Within-Accent Switch) | 3.75E-03 | 9.09E-03 | 0.41 | .68 |
| Accent (L2 Accent) : Switch (Across-Accent Switch) | -3.76E-02 | 8.62E-03 | -4.37 | < .001 |
| Accent (L2 Accent) : WARRM | 5.53E-03 | 5.19E-03 | 1.07 | .29 |
| Accent (L2 Accent) : Trail Making | 4.27E-03 | 4.69E-03 | 0.91 | .36 |
| Accent (L2 Accent) : Stroop | 1.02E-05 | 5.11E-04 | 0.02 | .98 |
| Switch (Within-Accent Switch) : WARRM | -1.06E-02 | 5.36E-03 | -1.97 | < .05 |
| Switch (Across-Accent Switch) : WARRM | -7.92E-03 | 5.09E-03 | -1.56 | .12 |
| Switch (Within-Accent Switch) : Trail Making | -2.40E-03 | 4.72E-03 | -0.51 | .61 |
| Switch (Across-Accent Switch) : Trail Making | -3.62E-05 | 4.50E-03 | -0.01 | .99 |
| Switch (Within-Accent Switch) : Stroop | -2.12E-05 | 5.29E-04 | -0.04 | .97 |
| Switch (Across-Accent Switch) : Stroop | 8.26E-05 | 5.03E-04 | 0.16 | .87 |

**Appendix B**

*Models from Analysis of Recognition Accuracy (Primary Task) Data*

**Table B1**

*Model with All Lower-Order Fixed Effects*

| **Predictor** | **Coefficient (𝛽)** | **S.E.** | ***z*** | ***p*** |
| --- | --- | --- | --- | --- |
| Intercept | 2.49 | 0.14 | 17.55 | < .001 |
| Accent (L2 Accent) | -2.45 | 0.04 | -68.5 | < .001 |
| Switch (Within-Accent Switch) | -0.04 | 0.05 | -0.96 | .34 |
| Switch (Across-Accent Switch) | -0.04 | 0.04 | -0.91 | .37 |
| WARRM | 0.09 | 0.02 | 3.57 | < .001 |
| Trail Making | -0.02 | 0.02 | -0.93 | 0.35 |
| Stroop | < 0.01 | < 0.01 | 1.31 | .19 |

**Table B2**

*Model with All Lower-Order Fixed Effects and Two-Way Interactions*

| **Predictor** | **Coefficient (𝛽)** | **S.E.** | ***z*** | ***p*** |
| --- | --- | --- | --- | --- |
| Intercept | 2.75 | 0.30 | 9.33 | < .001 |
| Accent (L2 Accent) | -2.08 | 0.21 | -9.77 | < .001 |
| Switch (Within-Accent Switch) | -0.86 | 0.27 | -3.17 | .002 |
| Switch (Across-Accent Switch) | -0.57 | 0.26 | -2.20 | .03 |
| WARRM | 0.03 | 0.06 | 0.53 | .60 |
| Trail Making | -0.09 | 0.05 | -1.84 | .07 |
| Stroop | 0.01 | 0.01 | 1.31 | .19 |
| Accent (L2 Accent) : Switch (Within-Accent Switch) | -0.10 | 0.11 | -0.85 | .39 |
| Accent (L2 Accent) : Switch (Across-Accent Switch) | 0.09 | 0.11 | 0.88 | .38 |
| Accent (L2 Accent) : WARRM | -0.07 | 0.04 | -1.68 | .09 |
| Accent (L2 Accent) : Trail Making | 0.05 | 0.04 | 1.46 | .14 |
| Accent (L2 Accent) : Stroop | -0.01 | < 0.01 | -1.92 | .06 |
| Switch (Within-Accent Switch) : WARRM | 0.19 | 0.05 | 3.43 | < .001 |
| Switch (Across-Accent Switch) : WARRM | 0.09 | 0.05 | 1.74 | .08 |
| Switch (Within-Accent Switch) : Trail Making | 0.08 | 0.05 | 1.70 | .09 |
| Switch (Across-Accent Switch) : Trail Making | 0.01 | 0.04 | 0.20 | .84 |
| Switch (Within-Accent Switch) : Stroop | < 0.01 | 0.01 | 0.20 | .85 |
| Switch (Across-Accent Switch) : Stroop | < 0.01 | 0.01 | 0.56 | .57 |

**Table B3**

*Model with All Lower-Order Fixed Effects, Two-Way Interactions, and Three-Way Interactions*

| **Predictor** | **Coefficient (𝛽)** | **S.E.** | ***z*** | ***p*** |
| --- | --- | --- | --- | --- |
| Intercept | 3.49 | 0.50 | 6.95 | < .001 |
| Accent (L2 Accent) | -3.03 | 0.55 | -5.51 | < .001 |
| Switch (Within-Accent Switch) | -2.01 | 0.56 | -3.59 | < .001 |
| Switch (Across-Accent Switch) | -1.20 | 0.54 | -2.21 | .03 |
| WARRM | -0.11 | 0.10 | -1.11 | .27 |
| Trail Making | -0.14 | 0.08 | -1.62 | .11 |
| Stroop | < 0.01 | 0.01 | 0.33 | .74 |
| Accent (L2 Accent) : Switch (Within-Accent Switch) | 1.40 | 0.63 | 2.21 | .03 |
| Accent (L2 Accent) : Switch (Across-Accent Switch) | 0.90 | 0.61 | 1.48 | .14 |
| Accent (L2 Accent) : WARRM | 0.12 | 0.11 | 1.04 | .30 |
| Accent (L2 Accent) : Trail Making | 0.11 | 0.09 | 1.17 | .24 |
| Accent (L2 Accent) : Stroop | < 0.01 | 0.01 | -0.22 | .83 |
| Switch (Within-Accent Switch) : WARRM | 0.42 | 0.12 | 3.55 | < .001 |
| Switch (Across-Accent Switch) : WARRM | 0.21 | 0.11 | 1.87 | .06 |
| Switch (Within-Accent Switch) : Trail Making | 0.15 | 0.10 | 1.49 | .14 |
| Switch (Across-Accent Switch) : Trail Making | 0.05 | 0.09 | 0.50 | .62 |
| Switch (Within-Accent Switch) : Stroop | 0.01 | 0.01 | 0.59 | .55 |
| Switch (Across-Accent Switch) : Stroop | 0.01 | 0.01 | 0.66 | .51 |
| Accent (L2 Accent) : Switch (Within-Accent Switch) : WARRM | -0.30 | 0.13 | -2.26 | .02 |
| Accent (L2 Accent) : Switch (Across-Accent Switch) : WARRM | -0.15 | 0.13 | -1.22 | .22 |
| Accent (L2 Accent) : Switch (Within-Accent Switch) : Trail Making | -0.09 | 0.11 | -0.78 | .43 |
| Accent (L2 Accent) : Switch (Across-Accent Switch) : Trail Making | -0.05 | 0.11 | -0.47 | .64 |
| Accent (L2 Accent) : Switch (Within-Accent Switch) : Stroop | -0.01 | 0.01 | -0.57 | .57 |
| Accent (L2 Accent) : Switch (Across-Accent Switch) : Stroop | -0.01 | 0.01 | -0.46 | .65 |
